# Poly[aqua­[μ_2_-*cis*-1,2-bis­(4-pyrid­yl)ethyl­ene-κ^2^ *N*:*N*′](μ_2_-5-nitro­isophthalato-κ^3^ *O*:*O*′,*O*′′)nickel(II)]

**DOI:** 10.1107/S1600536809053872

**Published:** 2009-12-19

**Authors:** Zhen-Zhong Fan, Guo-Ping Wang, Yu-Sheng Li

**Affiliations:** a Department of Petroleum Engineering, Daqing Petroleum Institute, Heilongjiang 151400, People’s Republic of China; b Department of Chemistry, Zhejiang University 310027, People’s Republic of China; c Second Oil Recovery Plant, Daqing Oilfields Co, Daqing 163414, People’s Republic of China

## Abstract

In the title compound, [Ni(C_8_H_3_NO_6_)(C_12_H_10_N_2_)(H_2_O)]_*n*_, the Ni^II^ atom is octa­hedrally coordinated by two *cis* N atoms from two different 1,2-bis­(4-pyrid­yl)ethyl­ene (bpe) ligands, two O atoms from one chelating carboxyl group of the 5-nitro­isophthalic acid (nip) ligand, one O atom from another monodentate nip ligand and one O atom from a water mol­ecule, forming a three-dimensional network structure. Inter­molecular O—H⋯O hydrogen bonding stabilizes this arrangement. The asymmetric unit of the structure contains one Ni^II^ atom, one water mol­ecule, one nip ligand and two half-mol­ecules of the bpe ligand with an inversion centre at the mid-point of the central C=C bond.

## Related literature

For structures containing nip ligands, see: Xiao & Yuan (2004 ▶); Xiao *et al.* (2005 ▶). For structures containing bpe ligands, see: Bauer & Weber (2009 ▶); Jung *et al.* (2009 ▶); Zheng & Zhu (2009 ▶).
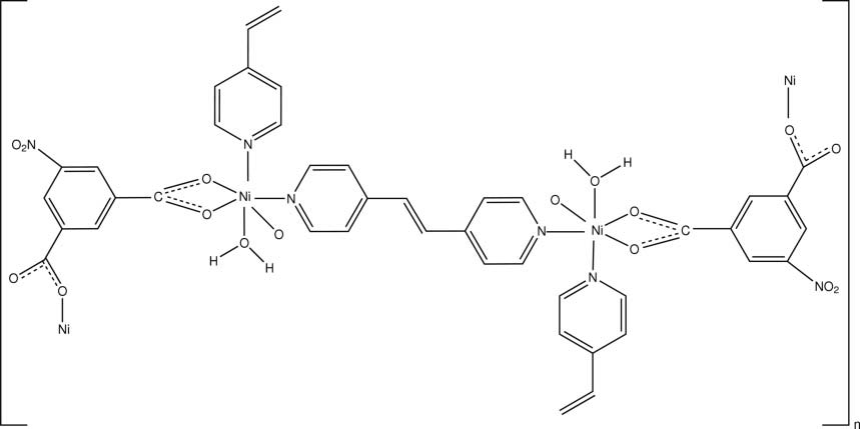


## Experimental

### 

#### Crystal data

[Ni(C_8_H_3_NO_6_)(C_12_H_10_N_2_)(H_2_O)]
*M* *_r_* = 468.06
Triclinic, 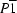

*a* = 9.3723 (6) Å
*b* = 10.9947 (7) Å
*c* = 11.1704 (8) Å
α = 109.970 (1)°
β = 90.190 (1)°
γ = 110.727 (1)°
*V* = 1001.68 (12) Å^3^
*Z* = 2
Mo *K*α radiation
μ = 1.02 mm^−1^
*T* = 293 K
0.43 × 0.24 × 0.15 mm

#### Data collection

Bruker SMART CCD area-detector diffractometer
Absorption correction: multi-scan (*SADABS*; Bruker, 2002 ▶) *T*_min_ = 0.669, *T*_max_ = 0.862
5380 measured reflections
3580 independent reflections
3332 reflections with *I* > 2σ(*I*)
*R*_int_ = 0.011

#### Refinement

*R*[*F* ^2^ > 2σ(*F* ^2^)] = 0.031
*wR*(*F* ^2^) = 0.089
*S* = 1.04
3580 reflections
287 parameters
1 restraint
H atoms treated by a mixture of independent and constrained refinement
Δρ_max_ = 1.05 e Å^−3^
Δρ_min_ = −0.29 e Å^−3^

### 

Data collection: *SMART* (Bruker, 2002 ▶); cell refinement: *SAINT* (Bruker, 2002 ▶); data reduction: *SAINT*; program(s) used to solve structure: *SHELXS97* (Sheldrick, 2008 ▶); program(s) used to refine structure: *SHELXL97* (Sheldrick, 2008 ▶); molecular graphics: *SHELXTL* (Sheldrick, 2008 ▶); software used to prepare material for publication: *SHELXTL*.

## Supplementary Material

Crystal structure: contains datablocks I, global. DOI: 10.1107/S1600536809053872/wm2289sup1.cif

Structure factors: contains datablocks I. DOI: 10.1107/S1600536809053872/wm2289Isup2.hkl

Additional supplementary materials:  crystallographic information; 3D view; checkCIF report

## Figures and Tables

**Table 1:** Selected bond lengths (Å)

Ni1—O3^i^	2.0326 (14)
Ni1—N1	2.0417 (17)
Ni1—N2	2.0777 (17)
Ni1—O7	2.0797 (15)
Ni1—O1	2.1031 (14)
Ni1—O2	2.2021 (14)

Symmetry code: (i) 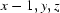.

**Table 2:** Hydrogen-bond geometry (Å, °)

*D*—H⋯*A*	*D*—H	H⋯*A*	*D*⋯*A*	*D*—H⋯*A*
O7—H7*C*⋯O4^i^	0.82	1.88	2.612 (2)	149
O7—H7*B*⋯O2^ii^	0.81 (1)	2.00 (1)	2.786 (2)	163 (3)

Symmetry codes: (i) 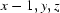; (ii) 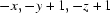.

**Fig. 1.**
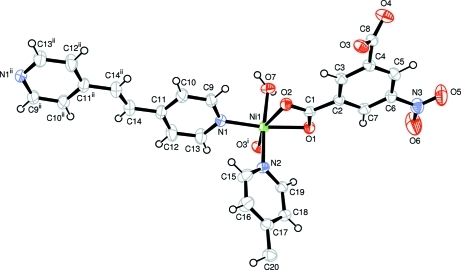
The coordination of the Ni (II) atom in the structure of the title compound, with atom labels and 50% probability displacement ellipsoids for all non-H atoms. [Symmetry codes: (i) x - 1, y, z; (ii) x + 1, y, z.]

**Fig. 2.**
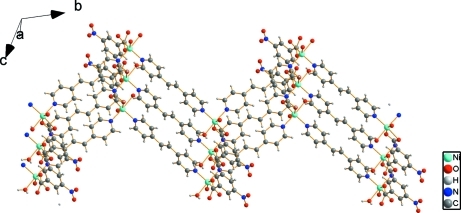
A part of the network structure of the title compound.

## supplementary crystallographic information

### Comment

Great interest has recently been focused on the crystal engineering of supramolecular architectures assembled by means of well designed organic ligands and metal ions under appropriate conditions. Previous reports have revealed that carboxylate ligands such as *m*-isophthalic acid can bind and bridge metal ions in various coordination modes, and bi-functional ligands, such as 4,4,-bipyridine or *trans*-1,2-bis(4-pyridyl)ethylene (bpe) also can link the metal ions to form network structures (Xiao & Yuan, 2004; Xiao *et al.*, 2005; Bauer & Weber, 2009; Jung *et al.*, 2009; Zheng & Zhu, 2009). Much less is known of systems containing two different ligands. Hence we have employed bpe and 5-nitroisophthalic acid (nip) as ligands in this work. We report herein the new three-dimensional structure of [Ni(nip)(bpe)(H_2_O)]_n_.

The asymmetric unit of the structure contains one Ni^II^ atom, one water molecule, one nip ligand and two half-molecules of the *N-*heterocycle with an inversion centre at the midpoint of the central C═C bond. The Ni1 site shows a slightly distorted octahedron with a NiN2O4 coordination set, as depicted in Fig.1, where the two equatorial N atoms (N1, N2) are from two different bpe ligands, four O atoms from two different nip (O1, O2 and O3^i^ atoms [symmetry code: (i) *x* - 1, *y*, *z*]), and one water molecule, respectively, with O2 and O3^i^ atoms [symmetry code: (i) *x* - 1, *y*, *z*] in the axial positions. The metal centres are connected in a three-dimensional fashion through the bpe and nip ligands. The Ni—O and Ni—N bond lengths fall in the ranges 2.0326 (14)–2.2021 (14) Å and 2.0417 (17)–2.0777 (17) Å, respectively. O—H···O hydrogen bonding between the water molecules and the O atoms of the free carboxylate groups stabilizes this assembly. The coordination water molecule forms strong hydrogen bonds O7—H7C···O4^i^ and O7—H7B···O2^v^ to the oxygen atoms of the carboxylate anion of the nip ligand (see Table 2). Fig. 2 shows a part of the packed structure of the title compound.

### Experimental

Nickel(II) acetate tetrahydrate (0.5 mmol), 5-nitroisophthalic acid (0.5 mmol) and 1,2-bis(4-pyridyl)ethylene] (0.5 mmol) were placed in a 30 ml teflon-lined, stainless-steel Parr autoclave together with water (20 ml). The autoclave was heated at 423 K for a week and was subsequently cooled slowly to room temperature. Green single crystals were obtained.

### Refinement

The H atoms of the water molecules were located in a difference Fourier map and were refined isotropically, with O—H and H—H distance restraints of 0.82 (1) Å and 1.37 (2) Å, respectively. There is a conspicious electron density of *ca* 1.1 electrons per cubic Angstrom at *ca x*=-0.27, *y*=0.5, *z*=-0.22. This points to a statistically disordered (s.o.f. *ca* 0.15) water molecule with distances of this highest peak to the H7B and O7 atoms of 2.78 Å and 3.11 Å, respectively. The remaining H atoms were positioned geometrically (C—H = 0.93 Å) and allowed to ride on their parent atoms. The *U*_iso_(H) values were set at 1.2*U*_eq_ (C) and 1.5*U*_eq_ (O).

### Crystal data

**Table:**

[Ni(C_8_H_3_NO_6_)(C_12_H_10_N_2_)(H_2_O)]	*Z* = 2
*M**_r_* = 468.06	*F*(000) = 480
Triclinic, *P*1	*D*_x_ = 1.552 Mg m^−^^3^
Hall symbol: -P 1	Mo *K*α radiation, λ = 0.71073 Å
*a* = 9.3723 (6) Å	Cell parameters from 563 reflections
*b* = 10.9947 (7) Å	θ = 2.3–25.2°
*c* = 11.1704 (8) Å	µ = 1.02 mm^−^^1^
α = 109.970 (1)°	*T* = 293 K
β = 90.190 (1)°	Prism, green
γ = 110.727 (1)°	0.43 × 0.24 × 0.15 mm
*V* = 1001.68 (12) Å^3^	

### Data collection

**Table:**

Bruker SMART CCD area-detector diffractometer	3580 independent reflections
Radiation source: fine-focus sealed tube	3332 reflections with *I* > 2σ(*I*)
graphite	*R*_int_ = 0.011
φ and ω scans	θ_max_ = 25.3°, θ_min_ = 2.0°
Absorption correction: multi-scan (*SADABS*; Bruker, 2002)	*h* = −10→11
*T*_min_ = 0.669, *T*_max_ = 0.862	*k* = −13→13
5380 measured reflections	*l* = −13→12

### Refinement

**Table:**

Refinement on *F*^2^	Primary atom site location: structure-invariant direct methods
Least-squares matrix: full	Secondary atom site location: difference Fourier map
*R*[*F*^2^ > 2σ(*F*^2^)] = 0.031	Hydrogen site location: inferred from neighbouring sites
*wR*(*F*^2^) = 0.089	H atoms treated by a mixture of independent and constrained refinement
*S* = 1.04	*w* = 1/[σ^2^(*F*_o_^2^) + (0.0584*P*)^2^ + 0.2791*P*] where *P* = (*F*_o_^2^ + 2*F*_c_^2^)/3
3580 reflections	(Δ/σ)_max_ = 0.001
287 parameters	Δρ_max_ = 1.05 e Å^−^^3^
1 restraint	Δρ_min_ = −0.29 e Å^−^^3^

### Special details

**Table:**

Geometry. All e.s.d.'s (except the e.s.d. in the dihedral angle between two l.s. planes) are estimated using the full covariance matrix. The cell e.s.d.'s are taken into account individually in the estimation of e.s.d.'s in distances, angles and torsion angles; correlations between e.s.d.'s in cell parameters are only used when they are defined by crystal symmetry. An approximate (isotropic) treatment of cell e.s.d.'s is used for estimating e.s.d.'s involving l.s. planes.
Refinement. Refinement of *F*^2^ against ALL reflections. The weighted *R*-factor *wR* and goodness of fit *S* are based on *F*^2^, conventional *R*-factors *R* are based on *F*, with *F* set to zero for negative *F*^2^. The threshold expression of *F*^2^ > σ(*F*^2^) is used only for calculating *R*-factors(gt) *etc*. and is not relevant to the choice of reflections for refinement. *R*-factors based on *F*^2^ are statistically about twice as large as those based on *F*, and *R*- factors based on ALL data will be even larger.

### Fractional atomic coordinates and isotropic or equivalent isotropic displacement parameters (Å^2^)

**Table:**

	*x*	*y*	*z*	*U*_iso_*/*U*_eq_	
Ni1	−0.10441 (3)	0.65566 (2)	0.40788 (2)	0.02504 (11)	
O1	0.08218 (16)	0.83742 (14)	0.52003 (14)	0.0310 (3)	
O2	0.12955 (16)	0.65335 (15)	0.40496 (14)	0.0317 (3)	
O3	0.72083 (17)	0.72162 (16)	0.46313 (14)	0.0335 (3)	
O4	0.7598 (2)	0.76834 (19)	0.67483 (15)	0.0452 (4)	
O5	0.7148 (2)	1.2514 (2)	0.8534 (2)	0.0669 (6)	
O6	0.4914 (3)	1.2513 (2)	0.8145 (3)	0.0922 (9)	
O7	−0.10853 (19)	0.59321 (17)	0.56426 (15)	0.0368 (4)	
H7C	−0.1478	0.6361	0.6203	0.055*	
N1	−0.2423 (2)	0.45995 (18)	0.28631 (17)	0.0310 (4)	
N2	−0.0886 (2)	0.73429 (18)	0.26156 (16)	0.0295 (4)	
N3	0.5841 (2)	1.1956 (2)	0.79624 (19)	0.0459 (5)	
C1	0.1744 (2)	0.7762 (2)	0.48722 (19)	0.0267 (4)	
C2	0.3389 (2)	0.8505 (2)	0.55081 (19)	0.0274 (4)	
C3	0.4422 (2)	0.7831 (2)	0.52789 (19)	0.0288 (4)	
H3	0.4126	0.6938	0.4655	0.035*	
C4	0.5902 (2)	0.8484 (2)	0.59767 (18)	0.0291 (4)	
C5	0.6353 (2)	0.9824 (2)	0.6881 (2)	0.0328 (5)	
H5	0.7322	1.0259	0.7372	0.039*	
C6	0.5339 (2)	1.0506 (2)	0.70426 (19)	0.0314 (4)	
C7	0.3861 (2)	0.9872 (2)	0.63851 (19)	0.0307 (4)	
H7A	0.3195	1.0348	0.6525	0.037*	
C8	0.6993 (2)	0.7724 (2)	0.57767 (19)	0.0296 (4)	
C9	−0.1887 (3)	0.3572 (2)	0.2488 (2)	0.0427 (6)	
H9	−0.0886	0.3762	0.2815	0.051*	
C10	−0.2739 (3)	0.2259 (2)	0.1648 (2)	0.0444 (6)	
H10	−0.2310	0.1581	0.1412	0.053*	
C11	−0.4237 (3)	0.1925 (2)	0.1143 (2)	0.0363 (5)	
C12	−0.4787 (3)	0.2997 (3)	0.1542 (2)	0.0475 (6)	
H12	−0.5788	0.2831	0.1235	0.057*	
C13	−0.3868 (3)	0.4297 (2)	0.2382 (2)	0.0436 (6)	
H13	−0.4263	0.4998	0.2628	0.052*	
C14	−0.5208 (3)	0.0535 (3)	0.0252 (2)	0.0428 (5)	
C15	−0.0938 (3)	0.6642 (2)	0.1362 (2)	0.0435 (6)	
H15	−0.1075	0.5704	0.1097	0.052*	
C16	−0.0798 (3)	0.7238 (3)	0.0449 (2)	0.0478 (6)	
H16	−0.0861	0.6701	−0.0412	0.057*	
C17	−0.0564 (3)	0.8640 (2)	0.0810 (2)	0.0354 (5)	
C18	−0.0545 (2)	0.9364 (2)	0.2110 (2)	0.0322 (5)	
H18	−0.0422	1.0300	0.2400	0.039*	
C19	−0.0710 (2)	0.8682 (2)	0.2961 (2)	0.0309 (4)	
H19	−0.0697	0.9182	0.3824	0.037*	
C20	−0.0330 (3)	0.9319 (2)	−0.0139 (2)	0.0388 (5)	
H20	−0.0673	0.8748	−0.1000	0.047*	
H14	−0.618 (3)	0.041 (3)	0.004 (3)	0.047*	
H7B	−0.133 (3)	0.5144 (15)	0.564 (3)	0.058*	

### Atomic displacement parameters (Å^2^)

**Table:**

	*U*^11^	*U*^22^	*U*^33^	*U*^12^	*U*^13^	*U*^23^
Ni1	0.02416 (16)	0.02304 (16)	0.02844 (16)	0.01047 (11)	0.00144 (10)	0.00846 (11)
O1	0.0250 (7)	0.0272 (7)	0.0388 (8)	0.0123 (6)	0.0017 (6)	0.0073 (6)
O2	0.0295 (8)	0.0274 (8)	0.0372 (8)	0.0142 (6)	0.0026 (6)	0.0072 (6)
O3	0.0311 (8)	0.0437 (9)	0.0329 (8)	0.0228 (7)	0.0069 (6)	0.0135 (7)
O4	0.0518 (10)	0.0629 (11)	0.0355 (8)	0.0384 (9)	0.0058 (7)	0.0179 (8)
O5	0.0436 (11)	0.0469 (11)	0.0771 (14)	0.0061 (9)	−0.0101 (10)	−0.0050 (10)
O6	0.0737 (16)	0.0553 (13)	0.114 (2)	0.0416 (12)	−0.0254 (14)	−0.0269 (13)
O7	0.0449 (9)	0.0370 (9)	0.0391 (8)	0.0229 (8)	0.0077 (7)	0.0189 (7)
N1	0.0319 (9)	0.0260 (9)	0.0316 (9)	0.0085 (7)	0.0016 (7)	0.0090 (7)
N2	0.0318 (9)	0.0297 (9)	0.0308 (9)	0.0145 (8)	0.0059 (7)	0.0125 (7)
N3	0.0435 (12)	0.0380 (11)	0.0462 (11)	0.0147 (10)	0.0013 (9)	0.0043 (9)
C1	0.0270 (10)	0.0270 (10)	0.0312 (10)	0.0130 (8)	0.0064 (8)	0.0139 (8)
C2	0.0252 (10)	0.0300 (10)	0.0301 (10)	0.0116 (8)	0.0048 (8)	0.0135 (8)
C3	0.0283 (10)	0.0301 (10)	0.0296 (10)	0.0135 (9)	0.0052 (8)	0.0103 (8)
C4	0.0246 (10)	0.0365 (12)	0.0318 (10)	0.0144 (9)	0.0082 (8)	0.0163 (9)
C5	0.0260 (11)	0.0387 (12)	0.0335 (11)	0.0120 (9)	0.0030 (8)	0.0130 (9)
C6	0.0305 (11)	0.0299 (11)	0.0312 (10)	0.0114 (9)	0.0052 (8)	0.0084 (9)
C7	0.0295 (11)	0.0317 (11)	0.0364 (11)	0.0168 (9)	0.0089 (9)	0.0137 (9)
C8	0.0238 (10)	0.0314 (11)	0.0346 (11)	0.0111 (9)	0.0036 (8)	0.0127 (9)
C9	0.0386 (13)	0.0323 (12)	0.0490 (13)	0.0135 (10)	−0.0102 (10)	0.0054 (10)
C10	0.0458 (14)	0.0309 (12)	0.0492 (14)	0.0160 (11)	−0.0063 (11)	0.0045 (10)
C11	0.0381 (12)	0.0295 (11)	0.0323 (11)	0.0057 (9)	0.0034 (9)	0.0079 (9)
C12	0.0296 (12)	0.0428 (14)	0.0534 (14)	0.0097 (10)	−0.0032 (10)	0.0020 (11)
C13	0.0328 (12)	0.0363 (12)	0.0517 (14)	0.0142 (10)	0.0003 (10)	0.0030 (11)
C14	0.0392 (13)	0.0359 (12)	0.0383 (12)	0.0055 (10)	−0.0005 (10)	0.0049 (10)
C15	0.0657 (17)	0.0315 (12)	0.0374 (12)	0.0226 (11)	0.0145 (11)	0.0130 (10)
C16	0.0717 (18)	0.0380 (13)	0.0313 (11)	0.0197 (12)	0.0149 (11)	0.0110 (10)
C17	0.0340 (12)	0.0387 (12)	0.0363 (11)	0.0129 (10)	0.0078 (9)	0.0178 (10)
C18	0.0343 (11)	0.0307 (11)	0.0354 (11)	0.0144 (9)	0.0061 (9)	0.0144 (9)
C19	0.0323 (11)	0.0306 (11)	0.0314 (10)	0.0144 (9)	0.0046 (8)	0.0107 (9)
C20	0.0433 (13)	0.0434 (12)	0.0324 (11)	0.0161 (10)	0.0059 (10)	0.0174 (10)

### Geometric parameters (Å, °)

**Table:**

Ni1—O3^i^	2.0326 (14)	C4—C8	1.507 (3)
Ni1—N1	2.0417 (17)	C5—C6	1.383 (3)
Ni1—N2	2.0777 (17)	C5—H5	0.9300
Ni1—O7	2.0797 (15)	C6—C7	1.380 (3)
Ni1—O1	2.1031 (14)	C7—H7A	0.9300
Ni1—O2	2.2021 (14)	C9—C10	1.363 (3)
Ni1—C1	2.470 (2)	C9—H9	0.9300
O1—C1	1.257 (2)	C10—C11	1.383 (3)
O2—C1	1.262 (2)	C10—H10	0.9300
O3—C8	1.258 (2)	C11—C12	1.388 (3)
O3—Ni1^ii^	2.0326 (14)	C11—C14	1.458 (3)
O4—C8	1.244 (3)	C12—C13	1.368 (3)
O5—N3	1.217 (3)	C12—H12	0.9300
O6—N3	1.210 (3)	C13—H13	0.9300
O7—H7C	0.8200	C14—C14^iii^	1.314 (5)
O7—H7B	0.814 (10)	C14—H14	0.89 (3)
N1—C9	1.335 (3)	C15—C16	1.372 (3)
N1—C13	1.337 (3)	C15—H15	0.9300
N2—C19	1.336 (3)	C16—C17	1.387 (3)
N2—C15	1.339 (3)	C16—H16	0.9300
N3—C6	1.471 (3)	C17—C18	1.394 (3)
C1—C2	1.501 (3)	C17—C20	1.468 (3)
C2—C3	1.390 (3)	C18—C19	1.377 (3)
C2—C7	1.391 (3)	C18—H18	0.9300
C3—C4	1.397 (3)	C19—H19	0.9300
C3—H3	0.9300	C20—C20^iv^	1.322 (5)
C4—C5	1.382 (3)	C20—H20	0.9300
			
O3^i^—Ni1—N1	95.70 (7)	C3—C4—C8	120.55 (18)
O3^i^—Ni1—N2	89.29 (6)	C4—C5—C6	118.92 (19)
N1—Ni1—N2	91.76 (7)	C4—C5—H5	120.5
O3^i^—Ni1—O7	90.31 (6)	C6—C5—H5	120.5
N1—Ni1—O7	92.91 (7)	C7—C6—C5	122.42 (19)
N2—Ni1—O7	175.33 (6)	C7—C6—N3	118.54 (19)
O3^i^—Ni1—O1	98.89 (6)	C5—C6—N3	119.04 (19)
N1—Ni1—O1	165.38 (6)	C6—C7—C2	118.61 (19)
N2—Ni1—O1	89.30 (6)	C6—C7—H7A	120.7
O7—Ni1—O1	86.16 (6)	C2—C7—H7A	120.7
O3^i^—Ni1—O2	159.73 (6)	O4—C8—O3	127.14 (19)
N1—Ni1—O2	104.19 (6)	O4—C8—C4	117.32 (18)
N2—Ni1—O2	93.84 (6)	O3—C8—C4	115.52 (17)
O7—Ni1—O2	84.95 (6)	N1—C9—C10	123.1 (2)
O1—Ni1—O2	61.19 (5)	N1—C9—H9	118.5
O3^i^—Ni1—C1	129.22 (6)	C10—C9—H9	118.5
N1—Ni1—C1	134.81 (7)	C9—C10—C11	120.5 (2)
N2—Ni1—C1	93.28 (7)	C9—C10—H10	119.7
O7—Ni1—C1	83.37 (6)	C11—C10—H10	119.7
O1—Ni1—C1	30.59 (6)	C10—C11—C12	116.1 (2)
O2—Ni1—C1	30.66 (6)	C10—C11—C14	123.0 (2)
C1—O1—Ni1	91.05 (12)	C12—C11—C14	121.0 (2)
C1—O2—Ni1	86.48 (11)	C13—C12—C11	120.5 (2)
C8—O3—Ni1^ii^	125.17 (13)	C13—C12—H12	119.7
Ni1—O7—H7C	109.5	C11—C12—H12	119.7
Ni1—O7—H7B	128 (2)	N1—C13—C12	122.6 (2)
H7C—O7—H7B	108.7	N1—C13—H13	118.7
C9—N1—C13	117.23 (19)	C12—C13—H13	118.7
C9—N1—Ni1	120.54 (15)	C14^iii^—C14—C11	126.5 (3)
C13—N1—Ni1	122.20 (15)	C14^iii^—C14—H14	117.9 (18)
C19—N2—C15	116.78 (18)	C11—C14—H14	115.6 (18)
C19—N2—Ni1	116.73 (14)	N2—C15—C16	123.3 (2)
C15—N2—Ni1	126.49 (15)	N2—C15—H15	118.3
O6—N3—O5	123.4 (2)	C16—C15—H15	118.3
O6—N3—C6	118.2 (2)	C15—C16—C17	120.0 (2)
O5—N3—C6	118.3 (2)	C15—C16—H16	120.0
O1—C1—O2	121.02 (18)	C17—C16—H16	120.0
O1—C1—C2	118.64 (18)	C16—C17—C18	116.7 (2)
O2—C1—C2	120.31 (17)	C16—C17—C20	121.1 (2)
O1—C1—Ni1	58.36 (10)	C18—C17—C20	122.2 (2)
O2—C1—Ni1	62.86 (10)	C19—C18—C17	119.4 (2)
C2—C1—Ni1	173.30 (14)	C19—C18—H18	120.3
C3—C2—C7	119.69 (19)	C17—C18—H18	120.3
C3—C2—C1	121.18 (18)	N2—C19—C18	123.63 (19)
C7—C2—C1	119.07 (18)	N2—C19—H19	118.2
C2—C3—C4	120.61 (19)	C18—C19—H19	118.2
C2—C3—H3	119.7	C20^iv^—C20—C17	124.9 (3)
C4—C3—H3	119.7	C20^iv^—C20—H20	117.6
C5—C4—C3	119.60 (18)	C17—C20—H20	117.6
C5—C4—C8	119.83 (18)		

Symmetry codes: (i) *x*−1, *y*, *z*; (ii) *x*+1, *y*, *z*; (iii) −*x*−1, −*y*, −*z*; (iv) −*x*, −*y*+2, −*z*.

### Hydrogen-bond geometry (Å, °)

**Table:**

*D*—H···*A*	*D*—H	H···*A*	*D*···*A*	*D*—H···*A*
O7—H7C···O4^i^	0.82	1.88	2.612 (2)	149
O7—H7B···O2^v^	0.81 (1)	2.00 (1)	2.786 (2)	163 (3)

Symmetry codes: (i) *x*−1, *y*, *z*; (v) −*x*, −*y*+1, −*z*+1.
